# Quinine a Cardiac Sedative in Hemorrhage

**Published:** 1875-02

**Authors:** 


Quinine as a Cardiac Sedative in Arresting Internal Hemorrhage.—Dr. S. Caro (*New York Medical Record*, June 1, 1874,) gives several examples in which, after all other remedies had failed, hæmoptysis, broncorrhea, epistaxis and menorrhagia were relieved by the use of quinine. To explain the results we make the following extracts: A solution of quinine—eight grains—was injected under the skin of the hind leg of a small dog. The temperature soon fell to four degrees. The chest being opened the heart was found relaxed, the cardiac impulse weakened, and the blood less rapid and voluminous. The arteries were seen to diminish in size, and the capillaries seemed empty or atrophied. In a second dog the same size fifteen grains were injected. The temperature was lowered eight degrees, and the animal soon died from paralysis of the heart. The chest being opened the heart was found completely relaxed, as soft as a rag, with scarcely any blood in the left ventricle. Into a third dog three grains were injected. The temperature rose one degree, and the pulse became more frequent. On opening the chest the heart was found tense and beating rapidly. Before the use of the quinine the blood corpuscles were found thin and running about; after the injection of the quinine the corpuscles were shrunken in various irregular shapes, indented or jagged at the edges, and very much altered in shape. A large number of observers believe that quinine acts upon the inter-cardiac nerves, producing an irritation of the peripheric extremity of the valves, paralyzing the peripheric extremity of the motor nerves of the heart, and the motor muscular system of the same, decreasing its activity and producing diminution of the blood pressure and peripheral heat. Cohnheim says that quinine has the power of arresting the motion of the white blood corpuscles, preventing the formation of animal heat and reducing blood pressure.

Bintz thinks that quinine in high doses paralyzes the action of the heart and arrests the motion of the white blood corpuscles by diminishing the oxidizing power of the red corpuscles, and in small doses acts like a tonic by increasing cardiac action. Traube says that the relaxed condition of the heart is produced by the stimulating action of the quinine upon the vagus.—*Detroit Review of Medicine and Pharmacy.*

